# Cranial shape comparison for automated objective 3D craniosynostosis surgery planning

**DOI:** 10.1038/s41598-018-21662-w

**Published:** 2018-02-20

**Authors:** Manon L. Tolhuisen, Guido A. de Jong, Ruud J. M. van Damme, Ferdinand van der Heijden, Hans H. K. Delye

**Affiliations:** 1 Department of Neurosurgery, Radboud University Medical Centre, Nijmegen, The Netherlands; 2 Department of Neurosurgery, Radboud University Medical Centre, Nijmegen, The Netherlands; 3 Faculty of Electrical Engineering, Mathematics and Computer Science, University of Twente, Enschede, The Netherlands; 4 University College Twente, Faculty of Electrical Engineering, Mathematics and Computer Science, University of Twente, Enschede, The Netherlands; 5 Robotics and Mechatronics, University of Twente, Enschede, The Netherlands; 6 Department of Neurosurgery, Radboud University Medical Centre, Nijmegen, The Netherlands

## Abstract

Virtual planning of open cranial vault reconstruction is used to simulate and define an pre-operative plan for craniosynostosis surgery. However, virtual planning techniques are subjective and dependent on the experience and preferences of the surgical team. To develop an objective automated 3D pre-operative planning technique for open cranial vault reconstructions, we used curvature maps for the shape comparison of the patient’s skull with an age-specific reference skull. We created an average skull for the age-group of 11–14 months. Also, we created an artificial test object and selected a cranial CT-scan of an 11 months old trigonocephaly patient as test case. Mesh data of skulls were created using marching cubes and raycasting. Curvature maps were computed using quadric surface fitting. The shape comparison was tested for the test object and the average skull. Finally, shape comparison was performed for the trigonocephalic skull with the average skull. Similar shapes and the area on the patient’s skull that maximally corresponded in shape with the reference shape were correctly identified. This study showed that curvature maps allow the comparison of craniosynostosis skulls with age-appropriate average skulls and a first step towards an objective user-independent pre-operative planning technique for open cranial vault reconstructions is made.

## Introduction

Craniosynostosis is the result of the premature fusion of one or more sutures within the new-born’s skull^1^. Patients of our medical centre with craniosynostosis that are older than 6 months are subjected to open cranial vault reconstruction. The goal of this treatment is to prevent or treat increased intracranial pressure by increasing the intracranial volume, but also to restore the cranial shape^2,3^. During the procedure, the cranial vault is divided in several osseous panels which are relocated and fixed to form a new cranial vault shape. Many different reconstruction techniques have been described for different forms of craniosynostosis and no consensus on the best technique exists^4–13^. Therefore, the location of cutting lines and the relocation of the panels is dependent on the experience and preference of the surgical team and the results will vary between surgical teams^14,15^.

With the use of a pre-operative plan for open cranial vault reconstruction, the reproducibility increases and the operation time, blood loss and infection rates decreases^16–18^. Current research focuses on the development of pre-operative planning techniques^16^^,^^17^^,^^19^. However, to our knowledge no solution has yet been found for the variance in results caused by subjective decision making. The developed techniques for the creation of a pre-operative plan still require interactive user-input, remain time consuming, complicated and labour-intensive, and follow an iterative process. Often several sessions between the medical technician and the surgeon are required to obtain a consensus on the feasibility of the created plan. No appropriate 3D reference data are available and the pre-operative plans are based on the opinion of the surgeon on what is beauty^16–19^.

The aim of this study is to set the first steps towards a user-independent automated pre-operative planning technique that fully eliminates subjectivity. This planning technique should merely consist of an objective evidenced-based algorithm allowing for the reconstruction to establish a shape of the patient’s cranial vault that maximally corresponds with an age-appropriate reference skull. We performed preliminary work on a method that enables the creation of average skulls that can be used as reference models within the pre-operative planning technique^20^. The current study focuses on an algorithm for the shape comparison of patients’ skulls with these reference models by identifying the region on the patient’s skull for which the shape maximally corresponds with the optimal shape of the reconstruction site.

## Methods and Materials

### Materials

The shape comparison algorithm was implemented in MATLAB 2016a^®^, The MathWorks, Inc., Massachusetts, U.S. OpenCL was used for GPU integration. A unit icosphere and a test object were created with 3ds Max 2016, Autodesk^®^, Inc., California, U.S. Twelve cranial CT-scans of patients within the age-group of 11–14 months and with no cranial pathology were used to create an average skull. Also, a pre-operative cranial CT-scan of an 11 months old trigonocephaly patient was used. All CT scans were obtained from the hospital database and provided as DICOM files. The use of the scans was approved by the committee on Medical Research Involving Human Subjects (CMO Arnhem-Nijmegen). In line with the Hospital research policy (opt-out system), patient consent was assumed as no patient objected to the use of his/her medical images. Thereby, all methods were performed in accordance with the national guidelines and regulations (Law on the Medical Contract and the Code on Good Medical Practice).

## Methods

### Comparative mesh data calculation

**Figure 1:**
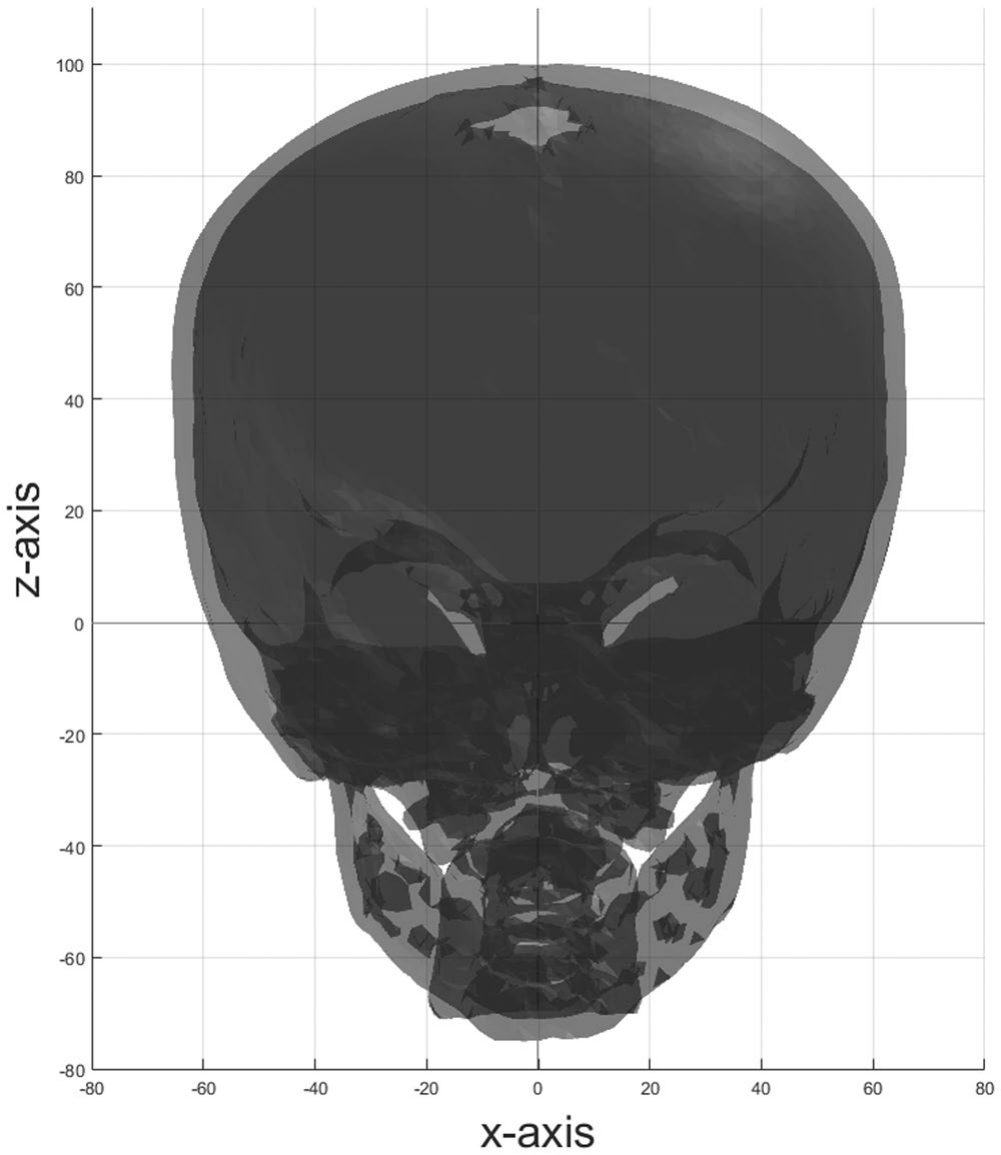
Inner and outer layer of a skull on which registration is applied.

**Figure 2:**
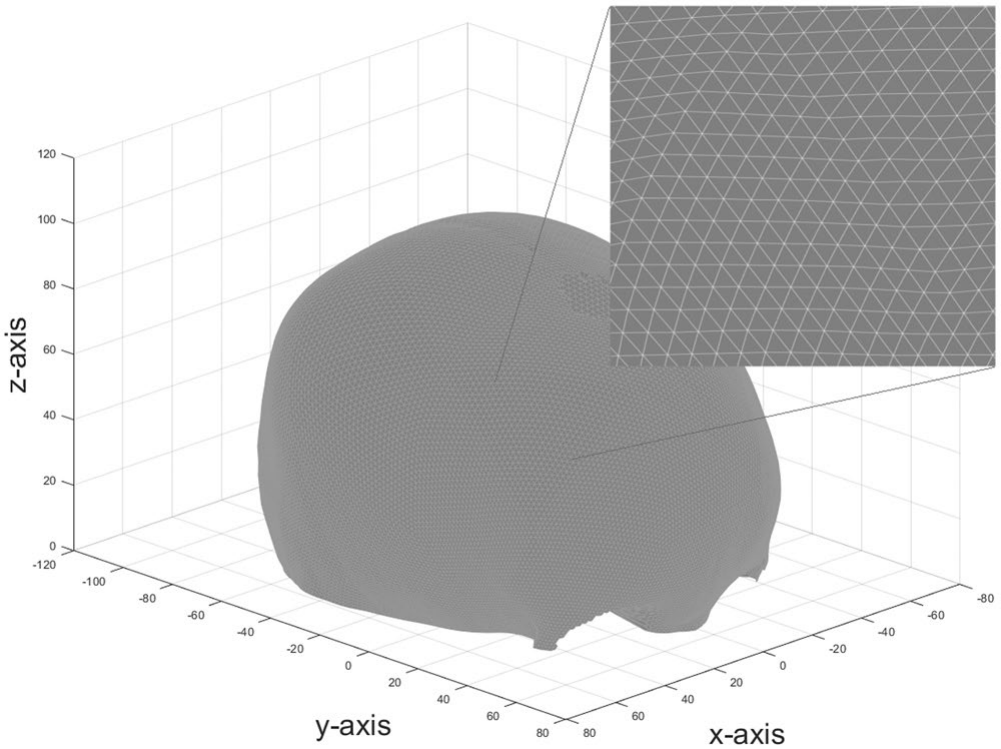
Sampled skull with a small area of the mesh data in more detail.

For each cranial CT-scan, the DICOM data were loaded and segmentation of the skull, based on a 167 HU threshold, was applied. Small isolated high intensity pixels were removed and a Gaussian filter was used to reduce noise in all slices. A marching cubes algorithm was used to create 3D surface mesh data^21^. Registration and resampling techniques were applied to all mesh data to similarly orient all skulls within a coordinate system and to create comparative data points. The centre of the sella turcica was placed at the origin of the coordinate system, the sella-nasion line was aligned onto the *y−*axis and both anterior clinoid processes were placed within the *x*,*y−*plane, such that the skulls were placed in a symmetrical straight-up position **(**Fig. 1**)**. Resampling of the mesh data was performed by raycasting based on a hemi-icosphere with 20641 vertices and 43284 faces. The directions of the rays were based on the vertices of the icosphere. The intersections of the rays with the inner and outer layer were found based on the Möller-Trumbore technique and stored separately^22^. Figure 2 shows that the raycasting approaches an even distribution of the data points on the surface of the skull. Because this study only focused on the shape comparison with no discrimination for fontanelles or holes within the skull, extrapolation of borders was used to fill in all the holes within the mesh data.

### Reference skull

The medical database was screened for CT-scans with no cranial pathology and twelve scans within the age-group of 11–14 months were found. All scans were processed according to the previous section. Since the vertex and face numbering for all skull mesh data was similar to the icosphere mesh data, the intersections of the rays with the inner and outer layer were suitable for averaging. The coordinate of intersection of each ray with the inner and outer layer was identified and averaged. This was performed for all scans. These coordinates formed the new coordinates of the inner and outer layer of the average skull for the respective vertex number. The shape of the average skull was used as reference for the presented 11 months old patient with trigonocephaly.

### Curvature estimation for mesh data

The Gaussian curvature of the skull was estimated for each vertex of the mesh data based on the quadric surface fitting method described by Hoppe *et al*.^23^ and Garland *et al*.^24^. For this, a quadratic function for each vertex was obtained that approximated the surface around the respective vertex. To compute the quadric surface, that is, the least square solution for the surface around a vertex, the quadrics of the faces within a specified radius from the vertex, weighted by their face area, were summed. Estimated curvatures depend on the spatial scale at which they are considered. This scale is directly linked to the neighbourhood radius of the surface patch on which the approximation is based. To select an appropriate neighbourhood radius, Gaussian curvature was estimated for the outer surface of the reference skull. The different neighbourhood radii were 15, 20, 25, 30 and 35 mm. The appropriate radius was selected based on detail discrimination. For each vertex of the mesh data, the quadratic functional was obtained and the Gaussian curvature was computed according to the method described by Goldman^25^.

Due to a large range in curvature values, clear representation by a colour map was challenging. Because a skull is spherical-like shaped and the Gaussian curvature (*k*_*G*_) of a sphere with radius *R*_*s*_ is defined as $\frac{1}{{R}_{s}^{2}}$, the curvature values were represented as $\frac{1}{\sqrt{{\kappa }_{G}}}={R}_{s}$ and further scaled using the logarithmic function: *sign*(*R*_*s*_)log(1+‖*R*_*s*_‖), as described in^26^. This enabled appropriate representation of the results by a colour map.

### Shape comparison

We used the method described by Gatzke *et al*.^26^ to quantify the shape correspondence of two areas based on curvature maps. Curvature maps were obtained for all vertices, based on 8-ring neighbourhoods. For every vertex, the mean Gaussian curvature for all vertices within every n-ring was computed and normalised to the surface of the ring. The same logarithmic scaling, as previously described for the colour maps, was applied to suppress high curvature ranges. By comparing curvature maps for different vertices the shape correspondence of different regions was determined. The correspondence of curvature maps is defined as the *L*_1_ difference between the discrete mean curvature values.

### Analysis

To verify the shape comparison algorithm, the method was performed for three test cases. First, shape comparison was applied on an artificial test object. The test object consisted of a sphere with 5 spherical bulges with different radii, a torus shaped bulge and a spherical hole. The reference area was selected at the top of the bulge with the second largest radius. The hole had the same radius as this bulge. Therefore, the shape of the interior surface of the hole was similar to the exterior shape of the bulge. The torus and hole were included in the test object to assess the discriminatory power of the curvature maps. The shape of the test object was compared with a 45° anti-clockwise rotated version of itself. The region on the rotated test object that showed the maximal shape correspondence with the reference area was determined.

Secondly, the shape comparison was applied within the reference skull. The right temple was selected as reference shape and the method was tested for the ability to identify the similar shape on the left temple.

Finally, the algorithm was tested for an 11 month old patient with trigonocephaly. Because, for trigonocephaly patients, the forehead is reconstructed, a reference area on the right side of the forehead of the reference skull was selected. The region on the trigonocephalic skull for which the curvature map maximally corresponded with the curvature map of the reference area was identified. Since only the shape of the outer surface of the skull is relevant for the aesthetics of the patient, the shape comparison was applied on only the outer layer of the skulls.

### Data availability

All relevant information for software implementation is described within this paper.

## Results

**Figure 3:**
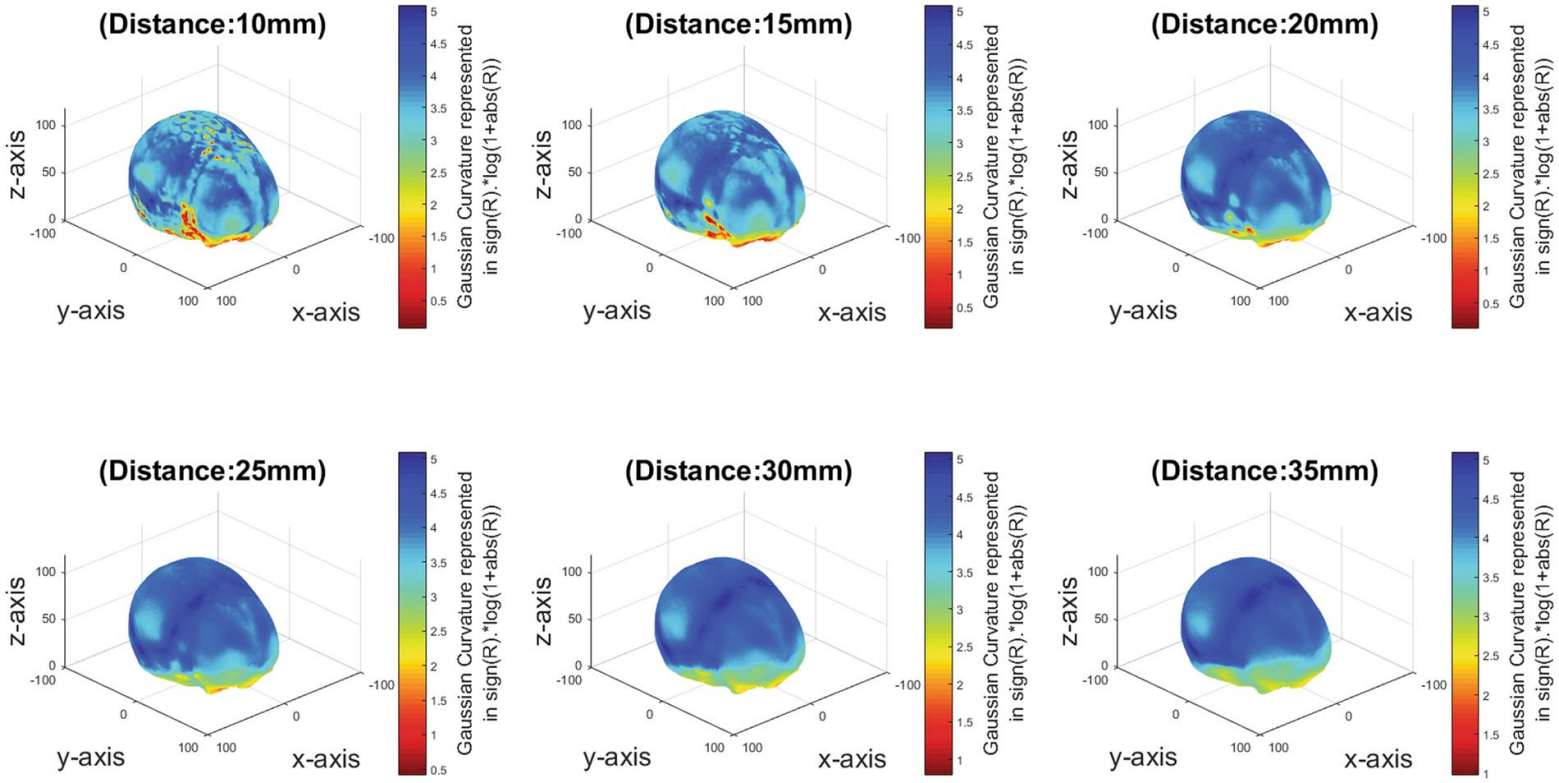
Curvature estimations for the reference skull with different neighbourhood radii (10, 15, 20, 25, 30, 35 *mm*).

Figure 3 shows the results of the curvature estimation for the reference skull for the neighbourhood radii of 10, 15, 20, 25, 30, and 35 mm. The neighbourhood radius of 15 mm appeared to produce the best results on detail discrimination. Hence, this was used for further analysis.

**Figure 4:**
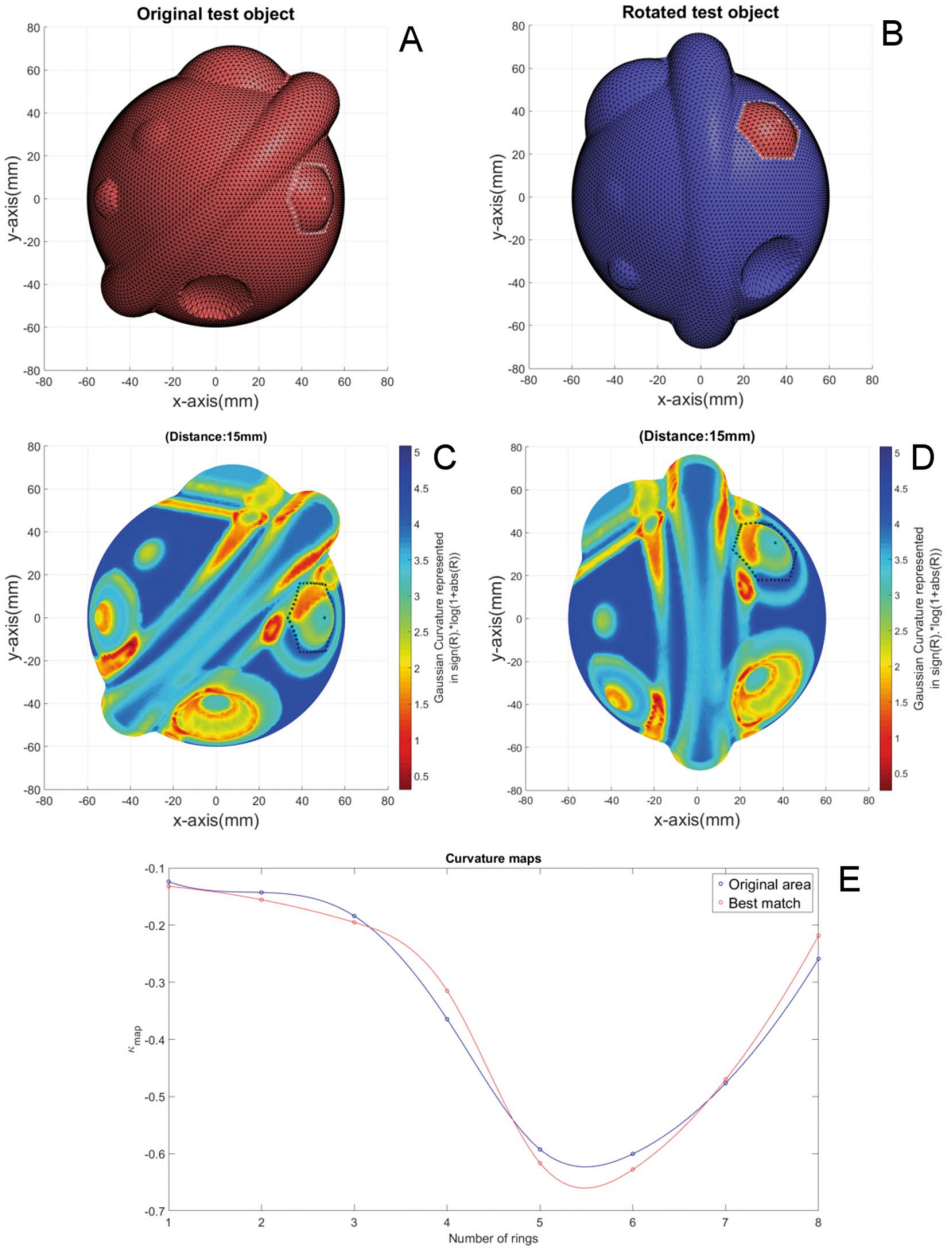
Shape comparison of the test object: (**A**) Original object. The border of the selected reference area and corresponding centre vertex are marked by the white dots. (**B**) The second object, which is a 45° degrees rotated version of the original. The area and corresponding centre vertex that were best matched with the reference area is represented in red and surrounded by white dots. (**C**) Curvature colour map of the original object. The border of the selected reference area and the centre vertex are marked by black dots. (**D**) Curvature colour map of second object. The border of the best matched area and the centre vertex are marked by black dots. (**E**) Curvatures maps of the reference area in blue and the matched area in red.

Figure 4(A,B) show that the curvature colour maps of both test objects were similar. Figure 4(A–D) show that the algorithm was able to find the area that matches the reference area. The curvature maps of the reference area and best matched area are shown in Fig. 4(E) and show similar patterns. The spherical bulges with different radii were not matched to the reference area. Also, the results show that the hole was discriminated from the bulge with the same radius.

**Figure 5:**
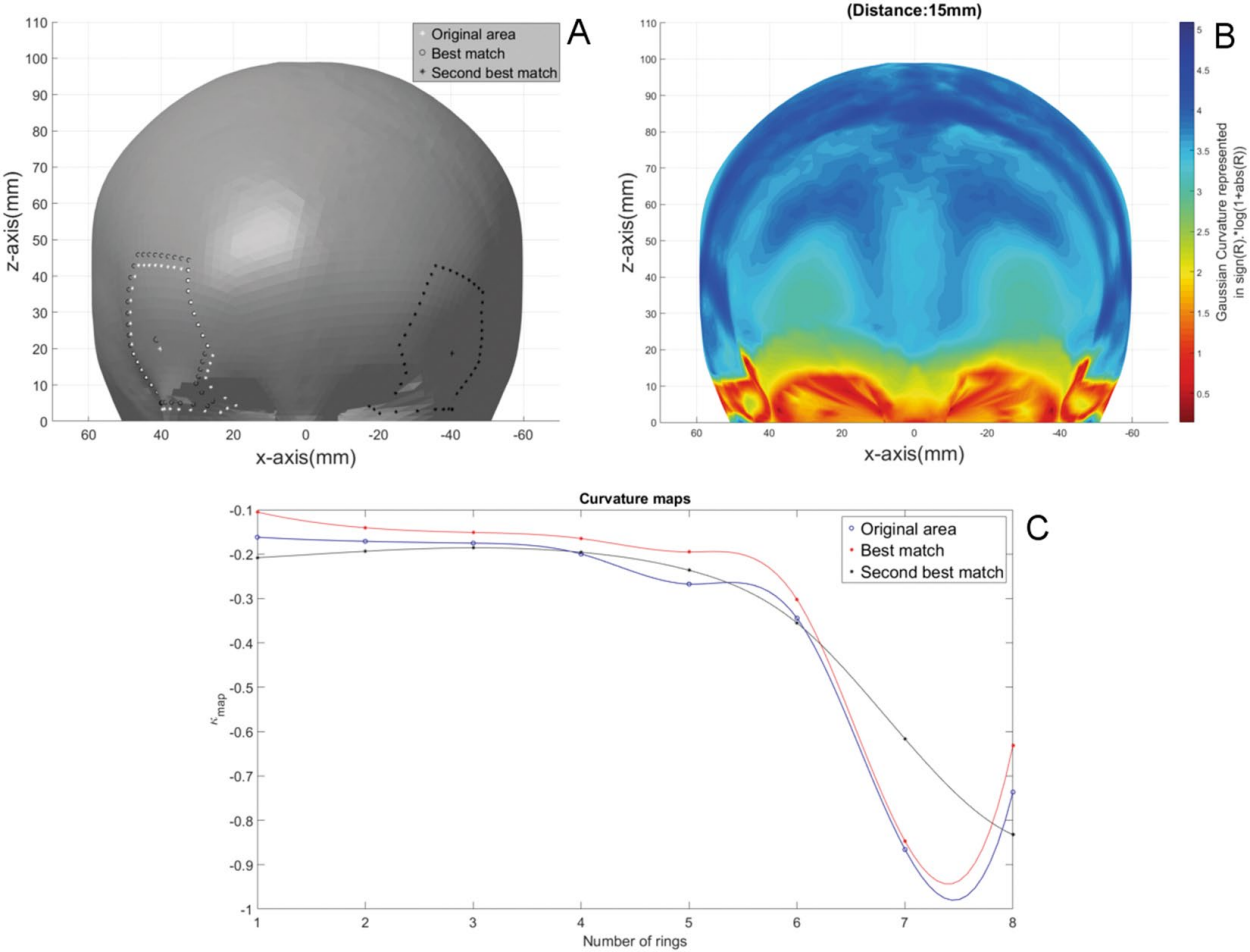
Shape comparison within a single skull: (**A**) The reference skull. The border of the selected reference area and the centre vertex are marked by white dots. The areas and centre vertices of the best match and second best match are marked by black circles and dots respectively. (**B**) Curvature colour map of skull. (**C**) Curvatures maps of the reference area in blue, the first matched area in red and the second matched area in black.

The curvature colour map in Fig. 5(B) confirms that the shape of the skull is symmetrical, with some subtile differences between left and right. The figure also shows the two best matched areas that were identified by the shape comparison. The best match was found close to the reference area. The second best match was the temple on the contralateral side of the reference area. Figure 5(E) show that the curvature maps of both matches contain similar patterns to the curvature map of the original area. Hereby, the curvature map of the best match shows more similarity to the reference than the second best match.

**Figure 6:**
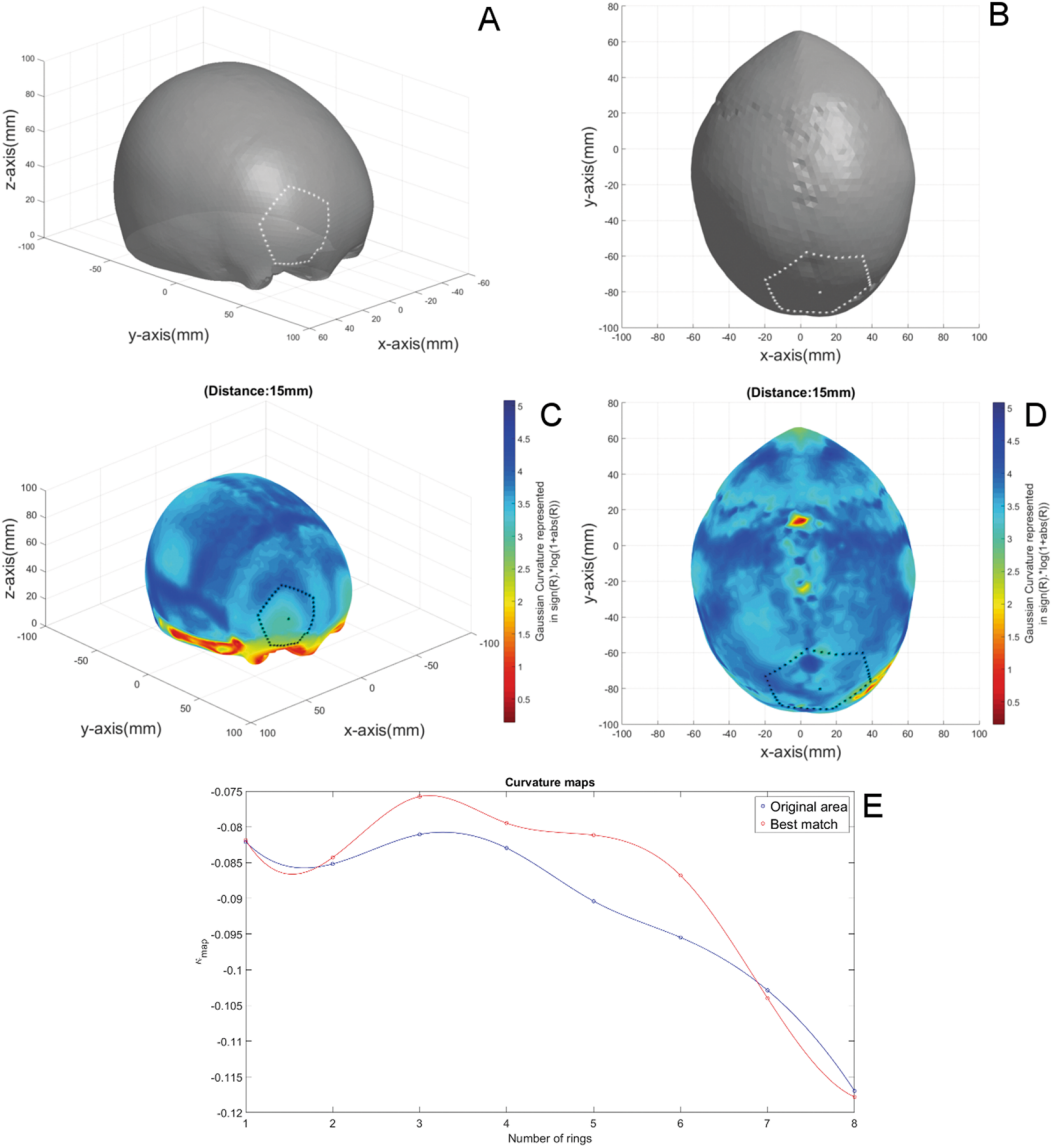
Shape comparison of a trigonocephaly with an age-appropriate reference skull: (**A**) Reference skull. The border of the selected reference area and corresponding centre vertex are marked by the white dots. (**B**) The trigonocephalic skull. The area and corresponding centre vertex that were matched with the reference area is surrounded by white dots. (**C**) Curvature colour map of the reference skull. The border of the selected reference area and the centre vertex are marked by black dots. (**D**) Curvature colour map of the trigonocephalic skull. The border of the best matched area and the centre vertex are marked by black dots. (**E**) Curvatures maps of the reference area in blue and the best matched area in red.

Figure 6 shows the results for the trigonocephaly case. The best match was found at the parietal bone of the trigonocephalic skull.

## Discussion

In this study, an algorithm was described that allows shape comparison of mesh data. This algorithm computes the gaussian curvature for all vertices of mesh data, based on quadric surface fitting, and creates a curvature map for each vertex by averaging the gaussian curvature of all vertices within an n-neighbourhood. Hereby, similar curvature maps correspond to similar shapes. The algorithm was able to find the corresponding region in a 45° anti-clockwise rotated version of the presented artificial test object. Also, the algorithm was able to find the contralateral region of a selected region on the reference skull, which was assumed to have a similar shape due to the symmetry of skulls. With these results, we showed that the algorithm is able to recognise similar shapes. Our future aim is to incorporate this algorithm within a fully automated objective pre-operative planning tool for open cranial vault reconstruction in craniosynostosis patients. We think that the algorithm can be used to find the optimal osseous panels, that would allow the surgeon to create a cranial shape that maximally corresponds with a reference shape.

We are well aware that most surgeons would apply some sort of overcorrection during surgical reconstruction to account for future growth in order to reach a ‘normal’ looking skull once growth is completed. Our algorithm can also be used to allow for overcorrection by scaling the reference skull with the desired degree of overcorrection before the comparison starts. We chose not to elaborate on this as the degree of overcorrection is surgeon-specific and we wanted to demonstrate the potential of our algorithm, without claiming the exact degree of overcorrection to be used.

The algorithm was tested for a trigonocephalic skull of an 11 months old patient. This is a common age for patients during open cranial vault reconstruction in our medical centre. The preliminary study clustered age-groups to compensate for the limited amount of scans found within the medical database. The age-groups were 2–4, 5–7, 8–10, 11–14, 15–18, 19–24, 25–36 and 37–48 months. The average skull from the age-group of 11–14 month was selected as age-appropriate reference model.

The neighbourhood radius of the curvature estimation was based on the reference skull. Rusinkiewicz *et al*.^27^ stated that the choice of the neighbourhood radius, e.g. the scale, on which the surface fitting is based is very important: where a small neighbourhood results in very accurate curvature estimation for clean data, increasing the neighbourhood size reduces the sensitivity for noise. The results confirmed this statement. Different neighbourhood radii should be used for skulls of different ages. Further evaluation on scaling is needed to determine the appropriate scaling required for different ages.

O’Neill^28^ describes that the Gaussian curvature directly provides information about the shape of a surface. A few challenges are encountered while computing the curvature of the skull. First, due to the mesh representation of the skulls, no parametric continuous closed function of the surface of the skull exists. The mesh is discrete data and provides no information about the surface around the specific point on which the curvature is computed. Therefore, the curvature of mesh data can only be estimated at a non-zero scale. Other challenges for curvature computation of mesh data include sampling, resolution, connectivity, holes and noise^27,29,30^. Several authors have drawn attention to curvature estimation methods on 3D mesh data^27,29–35^. According to the reviews of Gatzke *et al*.^29^ and Magid *et al*.^31^ multi-ring quadric surface fitting methods are the most accurate and robust for curvature estimation of mesh data. Therefore we decided to implement these methods and test the assumptions on several test objects.

The results on the artificial test object confirm that the shape comparison was able to identify areas with similar shape. The curvature colour maps showed corresponding curvature results, which is expected for objects with similar shape. However, the curvature map of the matched area contained small differences with the curvature map of the reference area. This difference was attributed to the estimation inaccuracy of the quadric surface fitting algorithm. Since the spherical bulges with different radii did not match, it is inferred that the curvature maps are scale variant. This is important for the planning technique, because ridges and bulges should be discriminated based on their size. Moreover, due to the similar radii of the hole and the reference area, the interior surface of the hole is similar in shape to the exterior surface of the bulge. Because it is not possible to use the interior surface of the cranium for the reconstruction, it is important that the shape comparison discriminates bulges from holes of similar shape. Our results show that the current algorithm is able to do so.

Furthermore, when testing the algorithm on a healthy skull, we expected that a shape on one side of the skull could also be found on the contralateral side of the skull, given the fact that a healthy skull is (almost) symmetrical. Indeed, the algorithm led to a best match that was almost the same area as the reference area. Because the second best match was found at the symmetrical region on the left side of the skull, it could be concluded that the shape comparison algorithm is able to identify corresponding shapes correctly.

For the trigonocephaly case, the area with the optimal shape for the selected reference area, the right side of the forehead, was located at the parietal bone. This result is in contradiction with the common practice, whereas literature only describes reconstruction techniques for trigonocephalic skulls that concern only the use of the frontal bone^5–8,12,13,17^. Because no appropriate age-specific 3D normative data was available till now, these techniques were originated from experience, subjective decision making (what is beauty?) and common practice. However, the results of this study were fully based on objective shape comparison with age-appropriate 3D averaged data and propose that, for the presented case, a totally different reconstruction technique could be considered. According to the results of this preliminary study, the parietal bone and not the contralateral frontal bone, would give rise to the ‘best’ frontal reconstruction of the presented trigonocephaly case. Albeit a first trial of the developed algorithm, this case shows clearly that the use of a fully objective automated pre-operative planning technique could be able to achieve better results by introducing out-of-the box solutions, based on reference data.

Currently, our algorithm merely performs shape analysis of the cranial vault and does not account for anatomical and surgical restrictions. Further developments should focus on the clinical feasibility of the reconstruction plan, limiting the morbidity, especially because the patient-group is very fragile. The reconstruction plan should avoid cutting lines near the superior sagittal sinus and sutures, and the use of cranial panels that are impossible to harvest from normal skull exposure. The example given in Fig. 6 would therefore never be a true surgical option. Jafarian *et al*.^36^ have developed an algorithm that automatically segments the separate osseous panels, sutures and fontanels of the new-born’s skull. Also, several vesselness filters are available, such as the Frangi filter^37^ or the Sato filter^38^, that are able to segment cranial vessels from CT-scans. The incorporation of these algorithms will allow the definition of a region of interest (ROI) for the shape comparison algorithm, that excludes cutting lines near the sutures and sinuses and osseous panels that are hard to harvest.

To guarantee the patient’s safety, the automated planning technique should create a pre-operative plan that will not only establish a cranial vault that maximally corresponds in shape with the age-appropriate reference model, but will also establish a stable structure. Plentiful and small reconstruction panels will conduct a better shape, but will hinder the stability of the structure. On the other hand, a small number of large panels will restrict the establishment of the optimal shape. Also, the thickness of osseous panels is a limiting factor for fixation, and thereby stability. Our future aim is to implement a cost function that finds the optimal panel shape, size and location, while constraining the number and location of osteotomies, based on anatomical constrictions, bone thickness and created structure stability.

## Conclusion

This study has implemented an algorithm for the shape comparison of skulls. Similar shapes were correctly identified and the identification of the area on a patient’s skull that maximally corresponded in shape with the reference shape was feasible. In conclusion, the algorithm can be used to determine the optimal osseous panels on the patient’s skull that allows the patient’s skull to be remodelled so that its shape maximally corresponds with the reference shape.
